# Selectively targeting BCL6 using a small-molecule inhibitor is a potential therapeutic strategy for glioblastoma

**DOI:** 10.1016/j.gendis.2025.101644

**Published:** 2025-04-15

**Authors:** Min Wu, Lin Zhang, Weikai Guo, Shiyi Lv, Wangrui Jin, Shuangshuang Zhu, Huang Chen, Shuyi Jian, Layang Liu, Yajing Xing, Shihong Peng, Mingyao Liu, Yihua Chen, Zhengfang Yi

**Affiliations:** a East China Normal University, Shanghai Key Laboratory of Regulatory Biology, Institute of Biomedical Sciences and School of Life Sciences, Shanghai 200241, China; b Changning Maternity and Infant Health Hospital, East China Normal University, Shanghai 200241, China; c School of Pharmaceutical Sciences and Yunnan Key Laboratory of Pharmacology for Natural Products, Kunming Medical University, Kunming, Yunnan 650500, China; d Yunnan College of Modern Biomedical Industry, Kunming Medical University, Kunming, Yunnan 650500, China; e Shanghai Yuyao Biotech Co., LTD., Shanghai 200241, China

**Keywords:** BCL6, Combination therapy, Glioblastoma, Temozolomide, YK01

## Abstract

Glioblastoma multiforme (GBM) is the deadliest form of brain tumor, and effective treatments are lacking. Thus, a new generation of effective treatments is urgently needed. B-cell lymphoma 6 (BCL6) is a transcription factor that functions to suppress the transcription of DNA damage response genes, halting cell death in response to DNA damage. Here, we identified BCL6 as a lynchpin in GBM, the expression of which was greater in GBM cells than in normal cells and associated with poor survival in GBM patients. The silencing of BCL6 additionally affected GBM cell proliferation and triggered cellular damage. Furthermore, we reported the identification of YK01, a novel small-molecule inhibitor of BCL6. YK01 exhibited excellent anti-GBM bioactivity and caused apoptosis; importantly, YK01 significantly inhibited the growth of GBM cells both *in vitro* and *in vivo*. Moreover, the combination of YK01 and temozolomide treatment significantly suppressed the growth and metastasis of tumors *in vivo* and prolonged the survival of mice with tumors. In summary, our findings reveal that BCL6 appears to play a crucial role in GBM and may be a therapeutic target for treating this incurable condition.

## Graphical abstract

**Figure:**
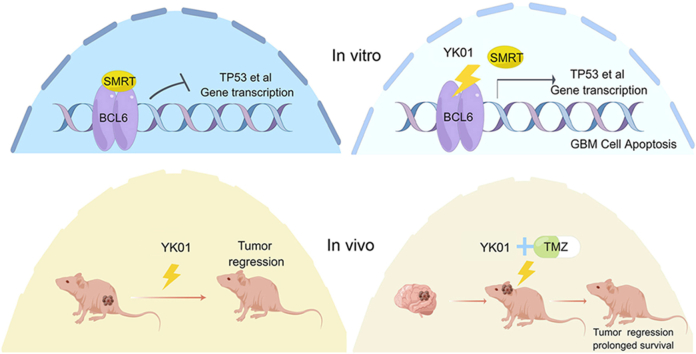

## Introduction

Glioblastoma multiforme (GBM) is the most common primary and refractory malignant tumor of the central nervous system and is associated with a high recurrence rate and mortality.^1^ The average survival time is only approximately 15 months.^2^ The WHO classifies glioma into grades 1–4 with increasing malignancy; grade 4 is further divided into several subtypes, such as isocitrate dehydrogenase (IDH) wild type and IDH mutant type, and IDH wild type accounts for approximately 90% of GBM cases.^3^^,^^4^ At present, GBM treatment mainly relies on surgical resection, radiotherapy, temozolomide (TMZ), and other adjuvant chemotherapies to prolong the survival of patients as much as possible, but the prognosis of patients is poor.^5^ Moreover, the limitations of TMZ chemotherapy are obvious in the clinic due to its toxic side effects, drug resistance, and the possibility of tumor recurrence after 8–9 months of treatment.^6^ Therefore, it is urgent to discover and develop specifically targeted therapies for GBM. The development of small-molecule drug-targeted therapies has always been a hot topic, but only a few breakthroughs have occurred due to the existence of the blood‒brain barrier.^7^ The development of small-molecule inhibitors for multiple targets, such as angiogenesis inhibitors, protease inhibitors, and inhibitors targeting epidermal growth factor receptors, has been limited in clinical trials.^8^ Some small-molecule drugs, such as paxalisib, which targets the phosphoinositide 3-kinase (PI3K)/protein kinase B (AKT)/mechanistic target of rapamycin (mTOR) pathway, have made some progress but are still in the early stage of research and development or clinical trials.^6^^,^^9^, ^10^, ^11^ To improve the treatment status of GBM, identifying new anti-cancer targets and developing targeted therapeutic methods are highly important.

B-cell lymphoma 6 (BCL6) is a proto-oncogene located on chromosome 3q27, also known as zinc finger and BTB domain containing 27 (ZBTB27), which was first identified in diffuse large B-cell lymphoma.^12^^,^^13^ As a transcriptional repressor, BCL6 inhibits a variety of cellular function regulatory genes, including ataxia telangiectasia and Rad3 related (ATR), tumor protein 53 (TP53), cyclin-dependent kinase inhibitor 1A (CDKN1A), and cluster of differentiation 69 (CD69). By recruiting three major transcriptional corepressors, SMRT (silencing mediator of retinoic acid and thyroid hormone receptor), NCOR (nuclear receptor corepressor), and BCOR (BCL6 corepressor), to bind to its N-terminal BTB domain, BCL6 is a key molecule in regulating the germinal center (GC) response during humoral immunity in the normal body.^14^, ^15^, ^16^ Failure to terminate BCL6 promptly while B cells are undergoing the GC response can lead to blocked terminal differentiation of B cells and ultimately drive the malignant phenotype of lymphoma.^17^ Recent studies have revealed that BCL6 plays a key role in a variety of tumors, such as acute lymphoid leukemia, chronic myeloid leukemia, breast cancer, and ovarian cancer, and these studies have implicated genomic amplification of the BCL6 locus in tumors.^17^, ^18^, ^19^, ^20^, ^21^, ^22^ In addition, BCL6 was found to be highly expressed in high-grade glioma, and its overexpression inhibited the apoptosis of glioma cells and became an essential factor for the survival of GBM cells. Moreover, the overexpression of BCL6 often predicts poor patient prognosis,^20^^,^^23^ and BCL6 is associated with GBM resistance. Inhibition of BCL6 activity facilitates increased sensitivity of GBM cells to TMZ and epidermal growth factor receptor (EGFR) inhibitors, which also has certain significance for combination therapy.^24^

Many BCL6 inhibitors that selectively kill tumor cells with high expression of BCL6 by blocking the interaction of BCL6 with SMRT, NCOR, and BCOR have been reported.^25^ However, the development of highly active BCL6 inhibitors is highly important for the treatment of patients with tumors. In our previous studies, we reported a series of BCL6 inhibitors with pyrimidinamine scaffold with good inhibitory activities against diffuse large B-cell lymphoma both *in vitro* and *in vivo*, but their BCL6 inhibitory activities remained at the micromolar level.^26^^,^^27^ More novel and efficient BCL6 inhibitors urgently need to be developed. Based on our previous work, we initiated a medicinal chemistry program aimed at modifying the structure of the pyrimidine diamine to further increase the inhibitory activity of BCL6, ultimately obtaining the highly effective BCL6 inhibitor YK01 with [1,2,4]triazoline [1,5-a] pyrimidine structural skeleton, and evaluated its biological functions and possible mechanism in inhibiting GBM. The results showed that YK01 inhibited the BCL6^BTB^/SMRT interaction and reactivated BCL6 target genes in a concentration-dependent manner, and YK01 inhibited GBM cell growth *in vitro* and *in vivo*. In addition, given the key role of BCL6 in tumor pressure resistance, our study has revealed that YK01 and TMZ have synergistic anti-GBM effects, which provides new strategies to improve GBM resistance.

## Materials and methods

### Mammalian cell culture

The HEK293T, U87-MG, U251, SF268, HS683, U118-MG, and SHG44 cell lines were purchased from the American Type Culture Collection. HEK293T were cultured in Dulbecco's modified Eagle medium (Gibco), with 10% fetal bovine serum (Invitrogen) and penicillin–streptomycin (Invitrogen). U87-MG, U251, SF268, HS683, U118-MG, and SHG44 were cultured in Dulbecco's modified Eagle medium (Gibco), with 10% fetal bovine serum (Invitrogen), glutamine (Invitrogen), sodium pyruvate (Invitrogen), and penicillin–streptomycin (Invitrogen). Culture conditions for cells were set at 37 °C and 5% CO_2_.

### Homogenous time-resolved fluorescence (HTRF) assay

BCL6-GST protein and SMRT-6His protein were first added to the 384-well plate (Greiner Bio-one, 784045), and then the compound to be tested was added in dilution buffer to incubate for 1 h, and then anti-6His-XL665 (Cisbio) and anti-GST-Tb (Cisbio) were added for overnight incubation. A microplate reader (BioTek Cytation5) read the values at 665 nm and 620 nm and recorded the data.

### Cell viability assay

Cell viability was tested by MTS assay (Promega, USA). 8000 cells were seeded in a 96-well plate and then treated with compounds for 72 h, followed by the addition of 20 μL of MTS and incubation at 37 °C for 2–3 h. Absorbance at 490 nm is measured with the SpectraMax 190 plate reader, and the IC_50_ value is calculated by GraphPad Prism. In the drug combination assay, combination index (CI) values were calculated using CalcuSyn software. A CI value greater than 1 has a drug antagonistic effect, and a value less than 1 has a synergistic effect.

### Quantitative reverse transcription PCR

RNA was extracted from U87-MG, U251, SF268, and HS683 cells treated with compounds for 24 h and reverse-transcribed into cDNA using Prime Script RT Kit (Takara). The cDNA was then used as a template for quantitative reverse transcription PCR reactions, and PCR reactions were performed with SYBR-Green (Takara).

### Animal immunity

The eight-week-old male C57BL/6 mice were immunized with antigen NP_18_-CGG, and each mouse was injected intraperitoneally with 100 mg. Two days later, the mice were treated with 50 mg/kg/d of FX1 or YK01 by intraperitoneal injection. The mice were euthanized after 12 days of continuous administration, and spleen and serum were collected. Flow cytometry was used to detect the proportion of GC-B cells in mouse serum. The serum was also subjected to enzyme-linked immunosorbent assay.

### Western blotting

Cell lines were treated with drugs, and tumor tissues were collected in a Ripa lysis buffer. Western blotting was performed using antibodies against BCL6 (14895S, Cell Signaling Technology), PARP (poly(ADP-Ribose) polymerase; 9532S, Cell Signaling Technology), γH2AX (phosphorylated form of H2A.X variant histone; 9718S, Cell Signaling Technology), phospho-ERK (4370S, Cell Signaling Technology), ERK (extracellular signal-regulated kinase; 4695T, Cell Signaling Technology), phospho-AKT (4060S, Cell Signaling Technology), AKT (9272S, Cell Signaling Technology), GAPDH (AB0036, ABWAYS), BCL2 (T40056F, ABWAYS), c-MYC (CY5150, ABWAYS).

### Xenograft tumor growth

The U87-MG xenograft tumor model was established by injecting 1 × 10^7^ cells mixed with Matrigel into female BALB/c nude mice (6–8 weeks old). When the tumor nodule volume reached 100 mm^3^, mice were randomized and treated with the indicated compound or vehicle intraperitoneally for 22 days. Tumor size was measured with a digital caliper, and tumor volume was calculated using the formula: volume = length × width^2^ × 0.52. After 22 days, all mice were sacrificed, and the primary tumor was excised and subjected to further analysis.

### Intracranial GBM xenografts and treatment

An orthotopic U87-MG GBM-bearing mouse model of BALB/c nude mice was created. Five microlitres of U87-MG-Luc cells (1 × 10^5^ cells) were implanted into the left striatum of anaesthetized animals. The burr hole was then filled with bone wax, and the scalp was closed with tissue glue. To assess the growth of the glioma, bioluminescence imaging (*in vivo* imaging system, Lumina III) was used to determine its size. The mice were treated with vehicle, TMZ (5 mg/kg, intraperitoneally), YK01 (25 mg/kg, intraperitoneally), or the combination of TMZ and YK01.

### Statistical analyses

*T*-tests were used in the statistical analysis. One-way ANOVA with Tukey's multiple comparison test was used to determine the differences between the control and experimental groups. Data were expressed as mean ± standard deviation, and *p*-values <0.05 were considered significant. All analyses were performed using GraphPad Prism 8 software.

## Results

### Identification of BCL6 as a growth-promoting factor in GBM

**Figure 1:**
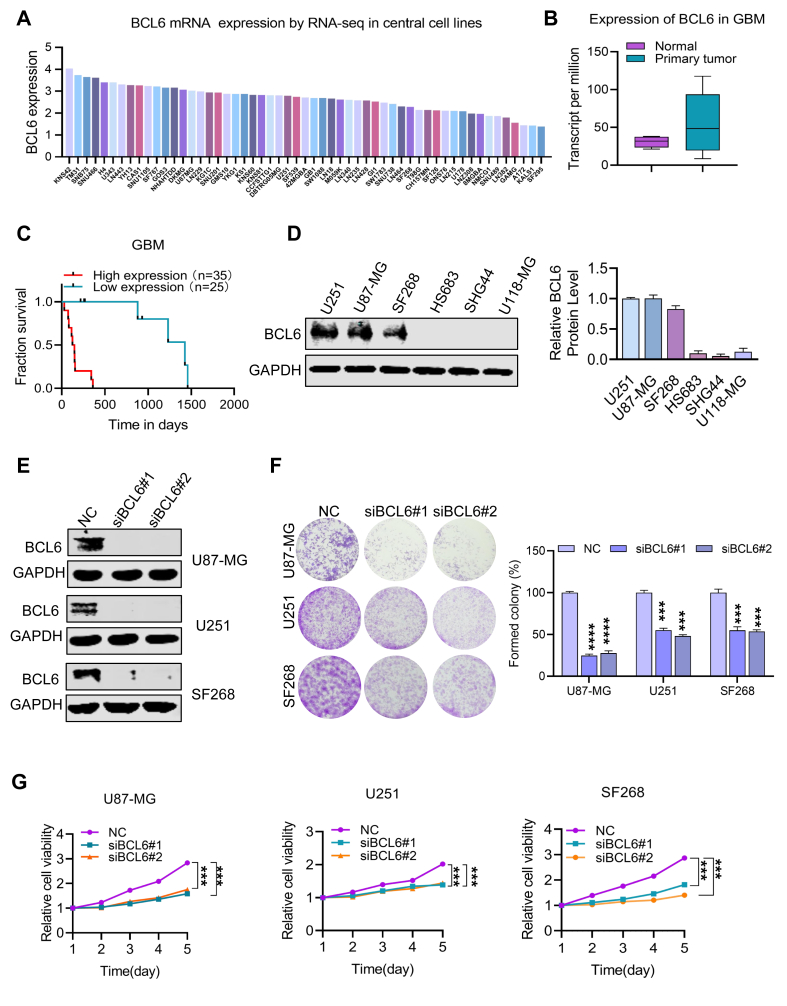
Identification of BCL6 as a growth-promoting factor in glioblastoma multiforme (GBM). **(A)** The expression level of BCL6 in glioma cell lines analyzed based on database data. **(B)** The expression of BCL6 in GBM patients analyzed using the TCGA database. **(C)** The relationship between BCL6 expression and survival of GBM patients analyzed using the TCGA database. **(D)** Immunoblots of BCL6 in the GBM cell lines U87-MG, U251, SF268, HS683, U118-MG, and SHG44. **(E)** Generation of scrambled siRNA (siNC and siBCL6) cell lines from U87-MG, U251, and SF268 cells. **(F)** Clonogenic assay of siNC and siBCL6 cell lines derived from U87-MG, U251, and SF268 cells. **(G)** Cell viability readouts of siNC and siBCL6 cell lines derived from U87-MG, U251, and SF268 cells. ∗∗*p* < 0.01, ∗∗∗*p* < 0.0001, and ∗∗∗∗*p* < 0.0001.

By analyzing the expression of BCL6 via RNA sequencing in central nervous system cells in the Cancer Cell Line Encyclopedia database and The Cancer Genome Atlas database, we found that the expression of BCL6 was relatively high in glioma cell lines (Fig. 1A; Fig. S1A). Moreover, BCL6 was overexpressed in GBM tumor samples compared with adjacent tissues according to database analysis (Fig. 1B). To elucidate the prognostic role of BCL6 in the survival of patients with GBM, normalized BCL6 expression data from 35 high-risk samples and 25 low-risk samples were downloaded from The Cancer Genome Atlas online database. The high-risk group was significantly correlated with increased BCL6 expression, suggesting that BCL6 overexpression was positively correlated with poor prognosis in GBM patients (Fig. 1C). In summary, abnormally high expression of BCL6 is associated with the occurrence, development, and prognosis of GBM. To further confirm the overexpression of BCL6 in glioma, we determined the expression level of BCL6 in glioma cells from multiple individuals through western blotting. The levels of BCL6 protein were significantly expressed in many GBM cells (Fig. 1D). To explore the biological effects of BCL6 in GBM cell lines, we established three BCL6-knockdown GBM cell lines via siBCL6 (Fig. 1E) and found that endogenous BCL6 deletion significantly reduced the viability and colony formation ability of the U87-MG, U251, and SF268 cell lines (Fig. 1F, G). After the overexpression of BCL6 in HS683 and SHG44 cells, we found that the overexpression of BCL6 increased the proliferation and colony formation abilities of the cells (Figs. S1B–G). These findings indicate that the survival of GBM cells is highly dependent on BCL6, suggesting that BCL6 may be a promising potential target for GBM therapy.

### YK01 is a small molecule inhibitor of BCL6^BTB^

**Figure 2:**
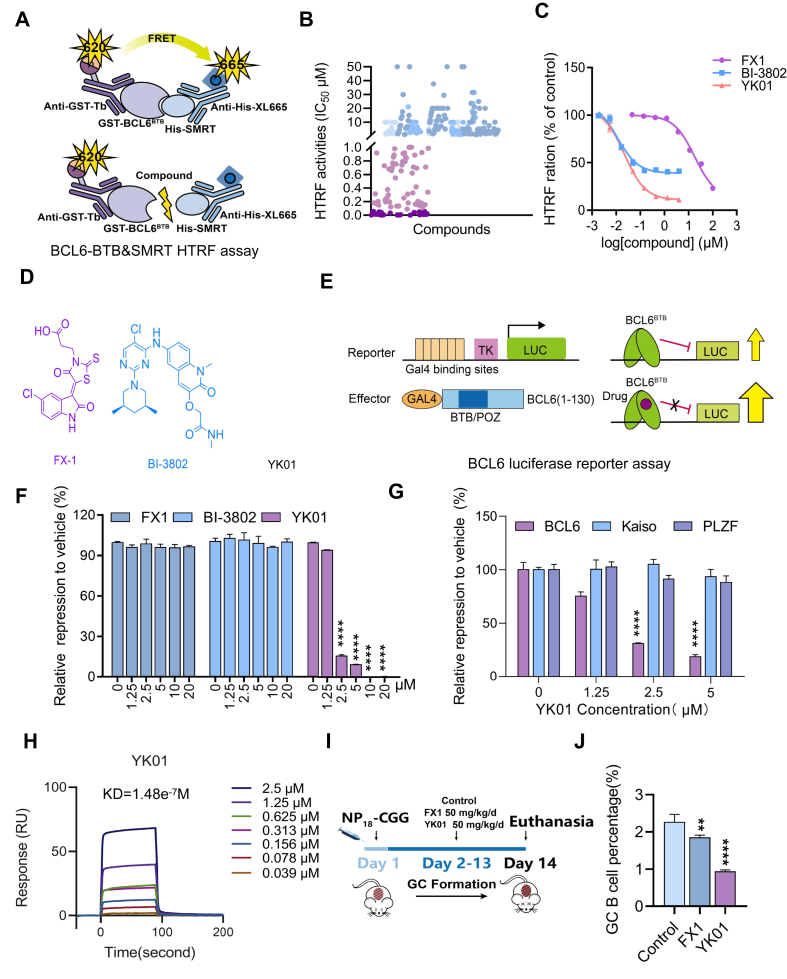
YK01 serves as a BCL6-BTB inhibitor. **(A)** Homogenous time-resolved fluorescence (HTRF) assay screening schematic. **(B)** HTRF assay screening compounds. **(C)** HTRF assay showed that the effect of FX1, BI-3802, and YK01 blocked the interaction between BCL6-BTB and SMRT. **(D)** Chemical structure of compound FX1, BI-3802, and YK01. **(E)** Schematic diagram of a luciferase reporter plasmid screening model. GAL4-DBD-BCL6-BTB bound to (GAL4)5-TK-LUC to inhibit fluorescence expression, and the fluorescence value recovered after the substance. **(F)** FX1, BI-3802, and YK01 inhibited BCL6-BTB-mediated transcriptional repression in luciferase reporter assays. **(G)** Reporter assays were performed to test the activities of YK01 with different BTB-related proteins (BCL6, Kaiso, and PLZF). **(H)** SPR sensorgram of YK01 binding to BCL6^BTB^, with YK01 concentrations and calculated K_D_ for binding shown. **(I)** C57/BL6 mice were immunized with NP_18_-CGG and intraperitoneally administered YK01 at a dosage of 50 mg/kg/d for 12 days to further detect whether YK01 could inhibit the transcriptional inhibitory function of BCL6 *in vivo*. **(J)** Flow cytometry detection of splenic GC-B cells (B220^+^GL7^+^FAS^+^) percentage and statistical graph of the total number of B cells or the percentage of GC-B cells in the mouse spleen. ∗∗*p* < 0.01 and ∗∗∗∗*p* < 0.0001.

In our previous work, we successfully identified a series of *N*-phenyl-4-pyrimidinamine BCL6 inhibitory activities against diffuse large B-cell lymphoma *in vitro* and *in vivo*. However, their BCL6 inhibitory activities remained at the micromolar level.^26^^,^^27^ To further improve the biological activities of BCL6 inhibitors, we employed scaffold hopping and bioisosteric replacement strategies to modify this structure. As a result, a novel class of [1,2,4]trizol[1,5-a]pyrimidine skeleton was designed and synthesized, and the BCL6 inhibitory activities of these compounds were evaluated via a HTRF system (Fig. 2A), and a batch of nM-level compounds were obtained (Fig. 2B; Fig. S2). The binding affinity of YK01 (IC_50_ = 11.7 nM), which was developed as a lead compound, was 3000-fold greater than that of the previously reported BCL6 inhibitor FX1 (IC_50_ = 37.68 μM) and comparable to that of the depressant BI3802 (IC_50_ = 2.7 nM) in the HTRF assay (Fig. 2C and D). To evaluate whether YK01 could inhibit BCL6-mediated transcriptional repression activity, a luciferase reporter assay was performed (Fig. 2E). YK01 significantly inhibited the transcriptional activity of BCL6^BTB^ in a concentration-dependent manner, and the transcriptional activity was almost completely disrupted at 2.5 μM, whereas FX1 and BI3802 had little effect on BCL6^BTB^ even at 20 μM (Fig. 2F). To determine whether YK01 may affect other BTB-containing proteins nonspecifically, PLZF and Kaiso, BTB zinc finger repressors belonging to the same family as BCL6, were tested as reporters in conjunction with BCL6 reporter assays. YK01 inhibited BCL6-BTB domain repression but did not affect the BTB domains associated with the Kaiso and PLZF proteins (Fig. 2G). To further verify whether YK01 directly bound to BCL6^BTB^, an SPR assay was conducted. YK01 directly bound to BCL6^BTB^ in a time-dependent saturation manner, with a K_D_ value of 148 nM (Fig. 2H). In summary, our results indicate that YK01 is a novel small-molecule inhibitor of BCL6^BTB^ that can directly bind to BCL6^BTB^ and inhibit its transcriptional activity.

BCL6 plays an important role in the formation of humoral immune GCs. To investigate whether YK01 inhibited BCL6^BTB^ *in vivo*, we examined the effect of YK01 on GC formation in mice (Fig. 2I). The results revealed that the proportion of GC-B cells in the YK01-treated group was significantly lower than that in the control group and FX1-treated group, suggesting that the function of BCL6 was inhibited *in vivo* but had little on the body weight of the mice *in vivo* (Fig. 2J; Fig. S3). In conclusion, the small molecule YK01 can directly bind to the BCL6-BTB domain and inhibit its biological functions *in vitro* and *in vivo*; thus, YK01 is a potential compound for the treatment of tumors with high expression of BCL6.

### YK01 inhibits the proliferation of glioma cells *in vitro*

**Figure 3:**
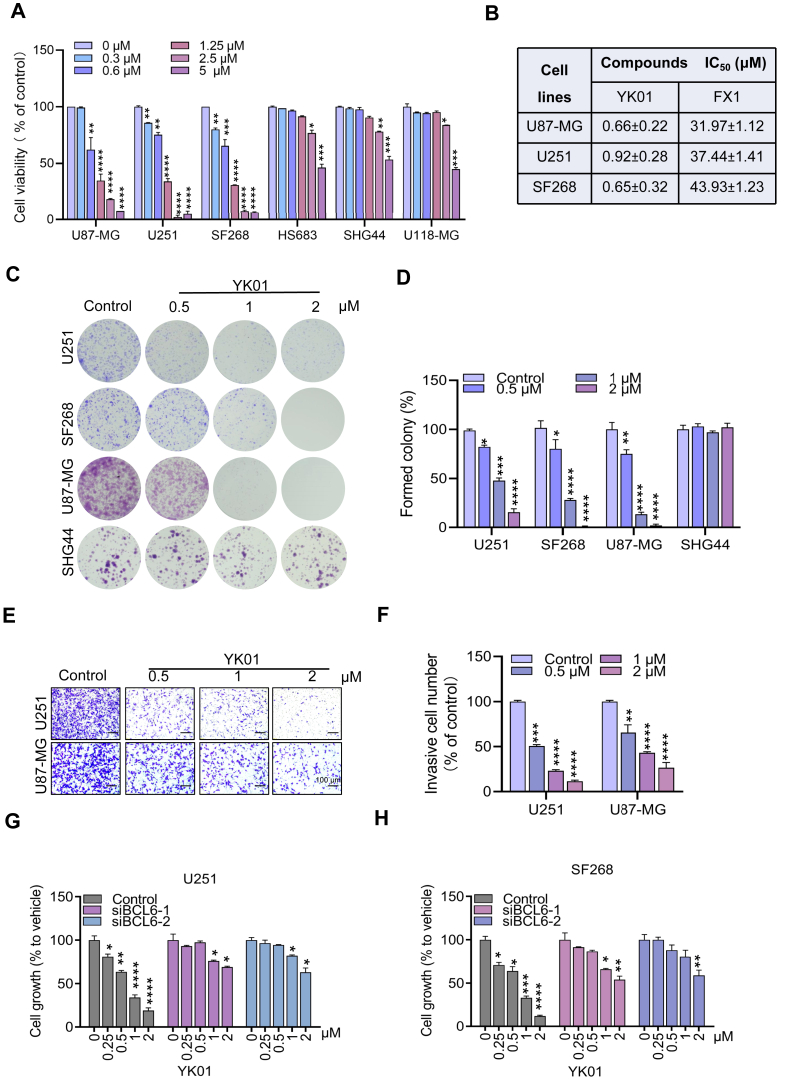
YK01 inhibits glioblastoma multiforme (GBM) cell proliferation. **(A)** MTS assays from 48 h YK01 treatment assessed the viability of multiple GBM cell lines. **(B)** IC_50_ values of YK01 and FX1 in GBM cells. **(C, D)** Clonogenic assay of U251, SF268, U87-MG, and HS683 cells treated with the indicated concentrations of YK01 for 7 days. **(E, F)** Anti-migration effects of YK01 on U251 and U87-MG after 24 h of treatment through transwell cell invasion assays. **(G, H)** Cell viability readouts of siNC and siBCL6 cell lines derived from U251 and SF268 cells with indicated concentrations of YK01 treatment for 72 h ∗*p* < 0.05, ∗∗*p* < 0.01, ∗∗∗*p* < 0.001, and ∗∗∗∗*p* < 0.0001.

To gain a deeper understanding of the role of YK01 in glioma cells *in vitro*, we performed MTS experiments and reported that YK01 significantly inhibited the proliferation of glioma cells with high BCL6 expression, which was almost 70-fold more potent than FX1 and almost 140-fold more potent than RI-BPI (Fig. 3A, B; Fig. S4). In addition, the capacities of YK01 to prevent colony formation and transwell invasion were measured. The results revealed that a low concentration of YK01 significantly inhibited clonal formation and invasion of glioma cells with high expression of BCL6 (Fig. 3C–F). To further verify that BCL6 is the primary target of YK01, U251 and SF268 cells with targeted BCL6 siRNA (siBCL6) were treated with YK01, and the corresponding cell viabilities were tested. We found that YK01 effectively inhibited the proliferation of control cells in a dose-dependent manner but had little effect on siBCL6 cells, indicating that YK01 inhibits GBM cell growth mainly by targeting BCL6 (Fig. 3G, H).

### YK01 reactivates BCL6 downstream genes and induces apoptosis in GBM cells

**Figure 4:**
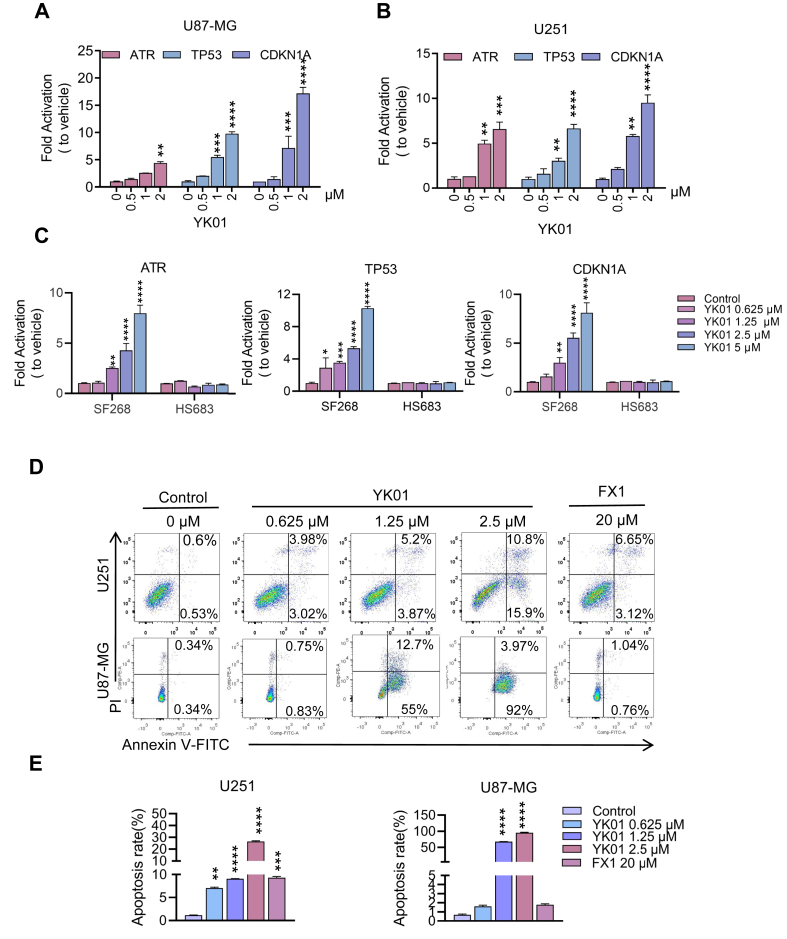
YK01 reactivates BCL6 target genes and induces glioblastoma multiforme cell apoptosis. **(A**–**C)** Changes in BCL6 target gene transcript abundance after 24 h YK01 treatment with the indicated concentrations in U87-MG, U251, SF268, and HS683 cell lines. **(D, E)** Apoptosis effects of U251 or U87-MG cells after treatment with FX1 and YK01 for 48 h *n* = 2. ∗*p* < 0.05, ∗∗*p* < 0.01, ∗∗∗*p* < 0.001, and ∗∗∗∗*p* < 0.0001.

To determine whether YK01 de-repressed BCL6 target genes in GBM cells, U87-MG and U251 cells were treated with YK01, and quantitative PCR experiments were conducted. In a concentration- and time-dependent manner, YK01 significantly reactivated the expression of BCL6 downstream genes, including ATR, TP53, and CDKN1A (Fig. 4A, B; Fig. S5). To explore whether the de-repression of the above genes induced by YK01 depended on BCL6, another BCL6-dependent GBM cell line (SF268) and a BCL6-independent GBM cell line (HS683) were further exposed to YK01. YK01 significantly up-regulated the expression of related genes in SF268 cells but had little effect on these genes in the HS683 cell line (Fig. 4C), demonstrating that YK01-induced de-repression of BCL6 target genes was dependent on BCL6. Annexin Y-FITC/PI double-staining was used to detect the apoptosis of GBM cells after treatment with YK01, and the results revealed that the apoptosis ratio of glioma cells increased significantly with the increase of YK01 concentration (Fig. 4D, E).

### YK01 induces the degradation of BCL6 and inhibits its ability to promote cancer

**Figure 5:**
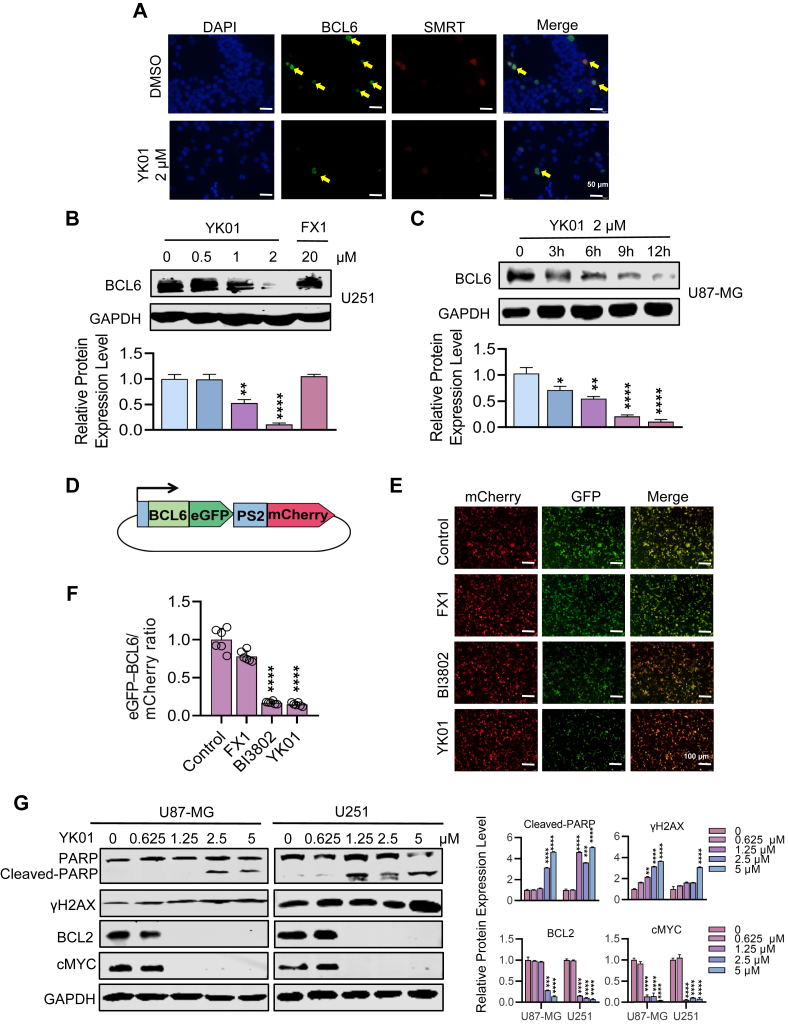
YK01 can induce the degradation of BCL6 protein and inhibit its function of promoting cancer. **(A)** YK01 blocks the colocalization of BCL6 and SMRT in U251 cell line. **(B)** After 24 h treatment with YK01 and FX1, western blotting detected the expression of BCL6 in U251 cells. **(C)** Western blotting detected the expression of BCL6 in U87-MG cells with YK01. **(D)** Schematic of the BCL6 stability reporter. **(E)** Immunofluorescence analysis of HEK293T cells expressing the BCL6-eGFP-mCherry reporter after treatment with DMSO, 10 μM FX1, 1 μM BI3802, or 1 μM YK01. **(F)** Fluorescence statistical analysis of BCL6-EGFP/mCherry. **(G)** The changes of DNA damage repair related proteins PARP, γH2AX, BCL2, and c-MYC with the concentration of YK01 were detected by western blotting after 24 h treatment of U87-MG and U251 cells.

To exert its biological effects, BCL6 needs to recruit corepressors such as SMRT to form a transcription complex, thereby inhibiting the expression of target genes.^15^ We demonstrated that YK01 disrupted the binding of BCL6^BTB^ and SMRT via an extracellular analysis by HTRF, but YK01 could also block the interaction between BCL6 and SMRT inside cells. To verify this, immunofluorescence staining was performed, and we found that YK01 blocked the colocalization of BCL6 and SMRT in U251 cells (Fig. 5A). YK01 treatment significantly reduced the expression of the BCL6 protein in cells (Fig. 5B, C). To explore whether the changes in BCL6 protein levels mediated by YK01 were related to protein degradation, we added CHX treatment, collected protein samples at 0 h, 2 h, 4 h, 8 h, and 16 h after the cells were treated, and performed western blotting assays to detect changes in the BCL6 protein. When protein synthesis was inhibited, the half-life (t_1/2_) of BCL6 protein degradation by YK01 was significantly reduced (Fig. S6). The above results show that YK01 can degrade the BCL6 protein. To further evaluate the ability of YK01 to induce BCL6 degradation, we generated a fluorescence reporter system in HEK293T cells containing a gene encoding the full-length BCL6 protein fused to eGFP, followed by the self-splintered polypeptides P2A and mCherry (Fig. 5D). As expected, YK01 or BI3802 treatment resulted in the degradation of eGFP-BCL6 (FL), whereas FX1 had little effect on the reporter gene (Fig. 5E, F). In addition, we examined changes in proteins involved in the DNA damage response and regulated by downstream targets of BCL6 after YK01 treatment of GBM cells. Previous studies have shown that GBM has abnormal PI3K-Akt and ERK signaling pathways, which promote the proliferation and survival of GBM. Western blotting results revealed that p-AKT, p-ERK, p-PI3K, p-p38, c-Myc, and the antiapoptotic protein BCL2 were significantly inhibited with increasing drug concentrations. Moreover, PARP cleavage and γH2AX expression increased with the increase of YK01 concentration (Fig. 5G; Fig. S7). Taken together, our data suggest that YK01 inhibits the activation of AKT and ERK and promotes the enhancement of the DNA damage response by blocking the ability of BCL6 to inhibit cell proliferation.

### YK01 inhibits tumor growth in GBM xenografts

**Figure 6:**
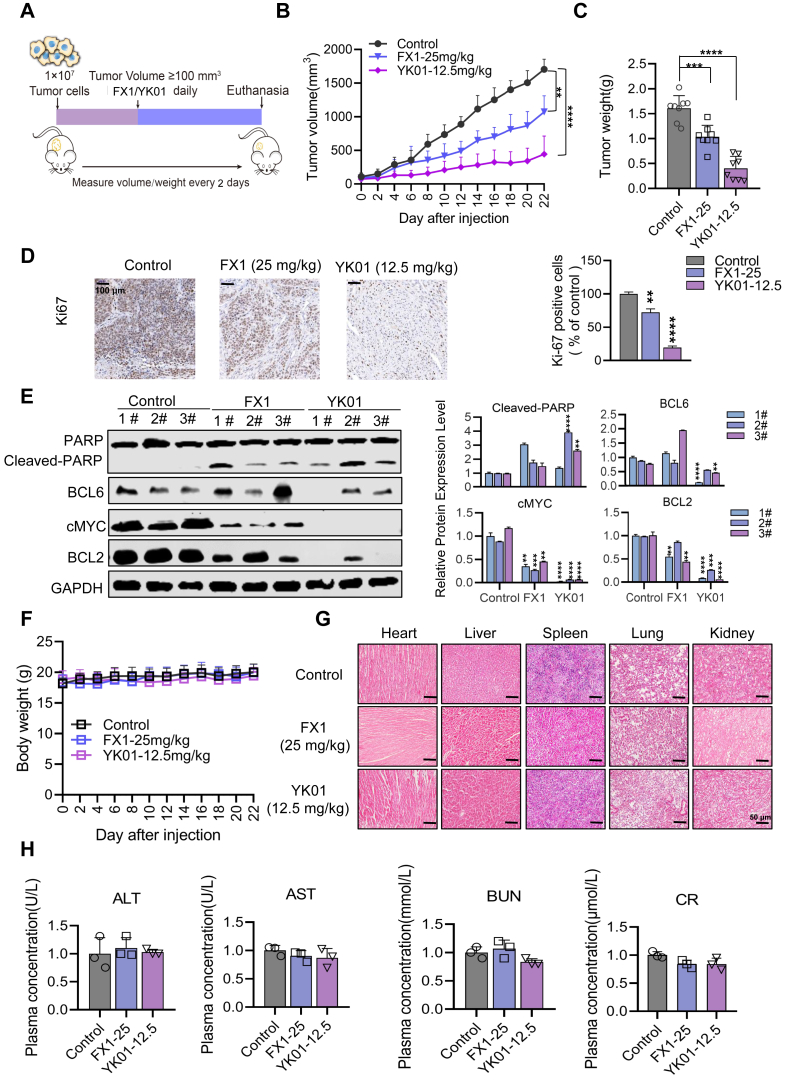
Anti-tumor effects of YK01 on U87-MG xenograft mouse models. **(A)** U87-MG xenograft mice were administered YK01 (12.5 mg/kg/d) and FX1 (25 mg/kg/d). **(B)** Tumor volumes (mm^3^) evaluated once every 2 days for a total of 22 days. **(C)** Tumors in each group. **(D)** Images of immunohistochemistry staining of Ki67. Scale bar = 50 μm. **(E)**Three tumor samples were selected from each of the four groups, and the expression of BCL6, PARP, c-MYC, and BCL2 were detected by western blotting. **(F)** Body weight changes of each group on day 21. **(G)** Hematoxylin-eosin staining of major organs of mice. Scale bars, 50 μm. **(H)** Liver function indexes, including aspartate aminotransferase (AST), alanine aminotransferase (ALT), blood urea nitrogen (BUN), and creatinine (CR) were detected. ∗*p* < 0.05, ∗∗*p* < 0.01, ∗∗∗*p* < 0.001, and ∗∗∗∗*p* < 0.0001. “n.s.” means no significance by log-rank (Mantel–Cox) test.

Our above data indicate that YK01 can significantly inhibit the proliferation and induce the apoptosis of GBM cells *in vitro*. However, its effect on anti-GBM growth *in vivo* is unknown. Therefore, a subcutaneous xenograft model was established by inserting U87-MG cells under the skin of female BALB/C-nude mice to evaluate the therapeutic effects of YK01 on GBM *in vivo*. There were three random groups of mice in which approximately 100 mm^3^ of tumor volume had formed: the carrier group, positive control group (FX1-25 mg/kg), and YK01 group (YK01–12.5 mg/kg) (Fig. 6A). As expected, YK01 significantly inhibited tumor growth compared with that of the control and was more potent than FX1 (Fig. 6B, C). The expression of the proliferative marker Ki67 was detected via immunohistochemical staining, and YK01 significantly inhibited the expression of Ki67 (Fig. 6D). In addition, the expression of proteins related to apoptosis, DNA damage repair, and BCL6 in different groups of tumor samples was detected via western blotting. Notably, the level of cleaved PARP was increased, whereas the levels of c-MYC, BCL2, and BCL6 were decreased compared with those in the control group, and YK01 had the greatest effect. These results indicated that the expression of tumor suppressor proteins was increased, the expression of tumor-promoting proteins was decreased in the drug administration group, and the protein changes in the YK01 group were relatively significant (Fig. 6E). To verify the safety of YK01 in mice, we examined the body weights of xenograft-bearing mice and found that YK01 had no significant effect on the body weights of the mice (Fig. 6F). Moreover, hematoxylin-eosin staining of major organ tissue slices revealed no morphological signs of toxicity in YK01 (Fig. 6G). After the end of drug treatment, three mice in each group were randomly selected for blood collection from their eyeballs, and common biochemical indicators in plasma, including blood urea nitrogen, creatinine, alanine aminotransferase, and aspartate aminotransferase, were tested. These indicators were all within the normal range, indicating that YK01 had no obvious toxicity or side effects. In summary, YK01 potently suppressed GBM growth *in vivo* without toxicity (Fig. 6H).

### YK01 and TMZ have synergistic inhibitory effects on GBM growth *in vitro* and *in vivo*

**Figure 7:**
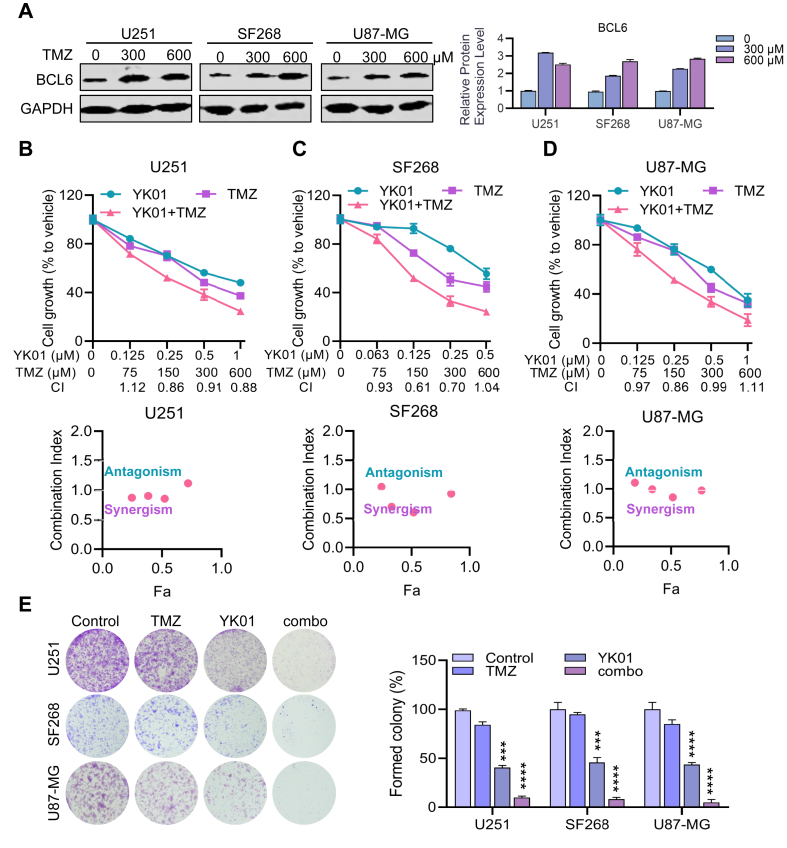
YK01 combined with temozolomide (TMZ) can synergically inhibit glioblastoma multiforme growth. **(A)** Expression levels of BCL6 were detected by western blotting following treatment of different concentrations of TMZ in U251, SF268, and U87-MG cells for 24 h. **(B**–**D)** Cell proliferation inhibition was detected by the combination of TMZ and YK01 in U251, SF268, and U87-MG cells for 24 h. The drug interaction coefficient (CDI) was calculated from the average survival rate. CDI < 1, CDI < 0.7, CDI = 1, and CDI > 1 indicated synergism, significant synergism, additivity, and antagonism, respectively. **(E)** Inhibition of clonogenic growth by the combined regimen.

Currently, most patients with GBM are treated with the alkylated chemical TMZ in addition to surgery and radiotherapy, but drug resistance and metastasis relapse lead to treatment failure.^28^ We aimed to determine whether the BCL6 inhibitor YK01 has a synergistic effect with TMZ. Western blotting revealed that BCL6 expression was up-regulated after treatment with TMZ, suggesting that the expression of BCL6 might be a factor contributing to TMZ resistance (Fig. 7A). Moreover, drug combination assays (CI values) were further employed to indicate drug synergy, and our results revealed that most CIs at different effective doses of each drug in GBM cell lines were all lower than a value of one, indicating the synergistic action of TMZ and YK01 (Fig. 7B–D). An analysis of the synergy plots of YK01 and TMZ revealed that the drug interaction in this combination was synergistic in the HAS models (Fig. S8). We further assessed the combined effects of YK01 and TMZ in a long-term colony formation assay. Our results showed that the combination of YK01 and TMZ led to robust growth inhibition of cultured colonies (Fig. 7E). An increased rate of apoptosis was observed in the combination drug group compared with that in the single-drug group (Fig. S9). These data suggest that the combination of the BCL6 inhibitor YK01 with TMZ can increase the sensitivity of GBM and partially overcome the protective effect of elevated BCL6 on GBM.

**Figure 8:**
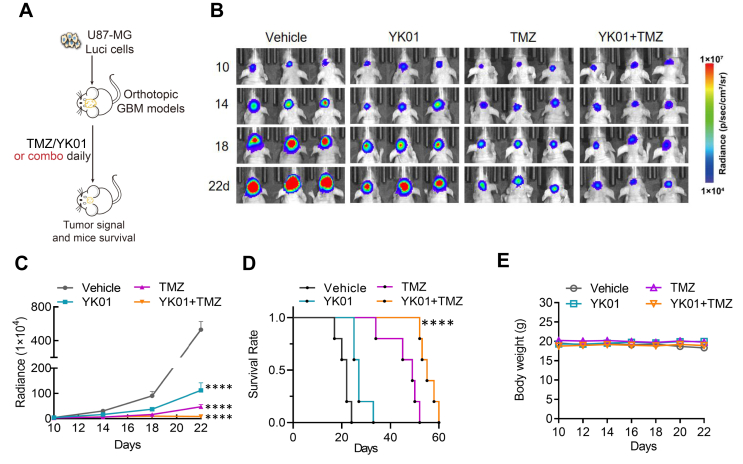
YK01 combined with temozolomide (TMZ) can synergically inhibit glioblastoma multiforme (GBM) growth *in vivo*. **(A)** Established primary GBM model using U87-MG cells expressing luciferase. **(B)** The growth of GBM xenografts was monitored by bioluminescence imaging on day 10, day 14, day 18, and day 22 after tumor implantation. **(C)***In vivo* bioluminescence images and quantification of tumor growth of U87-MG-Luci xenografts treated with TMZ, YK01, or vehicle after tumor implantation. **(D)** Kaplan–Meier survival analysis of mice bearing U87-MG-Luci xenografts treated with TMZ, YK01, or vehicle. *n* = 5 for each group. **(E)** Body weight changes of each group.

To investigate whether the inhibition of BCL6 could enhance the therapeutic effect of TMZ *in vivo*, we established a primary GBM model in which U87-MG cells expressed luciferase. A few days after the tumor cells were implanted, TMZ (5 mg/kg, intraperitoneally) with or without YK01 (12.5 mg/kg, intraperitoneally) and YK01 (12.5 mg/kg, intraperitoneally) alone were administered. Bioluminescence was used to monitor tumor growth signals via an *in vivo* imaging system (Fig. 8A). Bioluminescence analysis revealed that U87-MG-luci xenograft growth improved when the cells were treated with YK01 or TMZ alone, but YK01 combined with TMZ resulted in significantly better growth than TMZ alone (Fig. 8B, C). According to the Kaplan–Meier survival analysis, YK01 treatment had a relatively small effect on survival in the animals. However, the survival rate of YK01- and TMZ-treated mice was twice that of the TMZ-treated mice (Fig. 8D), confirming that YK01 enhanced the therapeutic effect of TMZ on the inhibition of GBM growth. To verify the safety of YK01 *in vivo*, we examined the body weight of the mice and found that YK01 had no significant effect (Fig. 8E). Taken together, YK01 is a BCL6 inhibitor that can effectively enhance the therapeutic effect of TMZ on GBM.

## Discussion

GBM is an aggressive tumor with a high degree of malignancy that is usually resistant to drug therapy and has a median survival of only 15 months.^29^ Currently, BCL6 translocations and overexpression have been observed in GBM patients, and the expression of BCL6 is associated with reduced apoptosis.^30^ In recent studies, the knockdown of BCL6 in tumor cells increased the expression of the proapoptotic proteins BAX and CDKN1A, which increased the activity of TP53 and reduced ERK activity.^6^^,^^21^ Tyrosine protein kinase receptors are transcriptionally regulated by BCL6 and decrease ERK activity following the inhibition of BCL6 and AXL in GBM. BCL6 plays a crucial role in cancer as a tumor suppressor. The structural properties of BCL6 make it a valuable target for the development of novel and selective anti-cancer therapies. A number of small molecules that inhibit peptides and BCL6 in cell and animal models of cancer have been developed. However, the solubility and activity of BCL6 inhibitors limit their further development, and no clinical studies have been conducted to date.^31^, ^32^, ^33^

In this study, we identified the key role of BCL6 in promoting the proliferation of glioma cells and the progression of glioma. Furthermore, the overexpression of BCL6 in GBM patients is associated with a poor prognosis. The mechanism underlying the overexpression of BCL6 in GBM is unclear, but our data suggest that the loss of BCL6 function can induce the apoptosis of GBM cells and inhibit their proliferation. In addition, we screened the selective small-molecule BCL6 inhibitor YK01 and evaluated its function and mechanism in inhibiting GBM. YK01 inhibited GBM cell growth *in vitro* and *in vivo* by inhibiting BCL6 to increase cell apoptosis and reduce proliferation. In a mouse subcutaneous glioma transplantation model, YK01 effectively inhibited tumor growth. In addition, given the key role of BCL6 in tumor pressure tolerance, our study revealed that YK01 and TMZ had synergistic anti-GBM effects, and the combination of YK01 effectively inhibited the *in situ* tumor growth of GBM and significantly prolonged survival. These results not only identify BCL6 as a growth-promoting factor for GBM development and YK01 as a potential drug for GBM treatment but also suggest a potential combination treatment strategy for GBM. Our study highlights the potential of BCL6 inhibition as a strategy to improve treatment outcomes in patients facing this adverse prognosis.

## CRediT authorship contribution statement

**Min Wu:** Writing – review & editing, Writing – original draft, Software. **Lin Zhang:** Validation, Software, Investigation. **Weikai Guo:** Validation, Software, Methodology. **Shiyi Lv:** Methodology, Investigation, Data curation. **Wangrui Jin:** Validation, Software. **Shuangshuang Zhu:** Supervision, Data curation. **Huang Chen:** Visualization, Methodology. **Shuyi Jian:** Software, Methodology. **Layang Liu:** Supervision, Software. **Yajing Xing:** Validation, Data curation. **Shihong Peng:** Validation. **Mingyao Liu:** Writing – review & editing. **Yihua Chen:** Writing – review & editing, Project administration. **Zhengfang Yi:** Writing – review & editing, Funding acquisition.

## Ethics declaration

All animals were obtained from the Animal Center of East China Normal University. The animal experiments have been approved by the Animal Investigation Committee of the Institute of Biomedical Sciences, East China Normal University.

## Data availability

The data that support the findings of this study are available from the corresponding author upon reasonable request. Some data may not be made available because of privacy or ethical restrictions.

## Funding

This work was supported by the grants from the National Natural Science Foundation of China (No. 82073310, 82373146, 82202897), The Jointed PI Program from Shanghai Changning Maternity and Infant Health Hospital (China) (No. PI202430), Shanghai Rising-Star Program (No. 23QB1405600), The Science and Technology Commission of Shanghai Municipality, China (No. 22QB1405600), Biomedical Projects of Yunnan Key Science and Technology Program (202502AA310002) and ECNU (East China Normal University) Construction Fund of Innovation and Entrepreneurship Laboratory (Shanghai, China) (No. 44400-20201-532300/021).

## Conflict of interests

The authors declared no competing interests.
