# Supplementary material for: Selectively targeting BCL6 using a small-molecule inhibitor is a potential therapeutic strategy for glioblastoma
Source: Genes Dis. 2025 Apr 15;12(6):101644. doi: 10.1016/j.gendis.2025.101644 (PMC12357248; doi:10.1016/j.gendis.2025.101644)
Supplement: Multimedia component 1 [file mmc1.docx]

Supplemental Materials for

**Selectively targeting BCL6 using a small-molecule inhibitor is**

**a potential therapeutic strategy for glioblastoma**

**Supplementary methods:**

***Surface plasmon resonance assay***

To test YK01 binding of BCL6^BTB^ protein, serially diluted concentrations of YK01 were injected into the flow system. Experiments were conducted using phosphate buffered saline (PBS) and the analyte was injected at the flow rate of 30 μL/min. The association time was 60 s and the dissociation time was 30 s. Since YK01 was dissolved in PBS containing 5% dimethyl sulfoxide and a solvent correction assay was performed to adjust the results. BCL6-BTB protein was immobilized on the sensor chip (CM5) using the amine-coupling method according to standard protocols. YK01 at various concentrations were injected into the flow system. The kinetics and affinity assay were examined at 25 ℃ at a flow rate of 30 μL/min. The K_D_ values were calculated with the kinetics and affinity analysis option of Biacore 8K evaluation software (version 3.0).

***Luciferase reporter assay***

The reporter construct (GAL4)5-TK-LUC and the GAL4-DBD-BCL6BTB, GAL4-DBD-KaisoBTB and GAL4-DBD-PLZFBTB expression plasmid were kindly provided by Dr. Ari Melnick (Weill Cornell Medical College, Department of Haematology/Oncology, New York, NY, USA). HEK293T cells were co-transfected with (GAL4)5-TK-LUC and GAL4-DBD-BCL6BTB or GAL4-DBD-KaisoBTB, GAL4-DBD-PLZFBTB and GAL4-DBD plus a TK-Renilla reporter construct using Lipofectamine 3000 (Thermo Fisher Scientific). After 6 h of transfection, cell lysates were collected for value measurement after 24 h of treatment of cells with the compound. TK-Renilla luciferase plasmid was used as an internal control (Promega, WA, USA). Dual luciferase assays were performed according to the manufacturer's guidelines (Promega, WA, USA).

***Flow Cytometric Analysis***

Single-cell suspensions were prepared and red blood cells were lysed by ACK. Antibodies used for staining were PerCP/cy5.5-anti-B220 (Biolegend, 103236), FITC-anti-FAS (BD Pharmingen, 554257), eFluor660-anti-GL7 (Invitrogen, 50- 5902-82), PerCP/cy5.5-anti-CD4 (BioLegend, 100434), PE-antiCXCR5 (BioLegend, 145504), and APC-anti-PD1 (BioLegend, 109112). The stained cells were analyzed using a FACS Calibur (BD).

***Cell Apoptosis Assay***

GBM cells were cultured in a 6-well plate with different concentrations of compounds for 48 h. Cells were collected and washed with PBS, then re-suspended in binding buffer, and incubated with Annexin V-FITC and propidium iodide. About 15 min later, cells were analyzed immediately with flow cytometry (FACS Calibur, BD Biosciences).

**Figure S1**. **Identification of BCL6 as a growth promoting factor in GBM .**

**(A)** Analyze the mRNA expression level of BCL6 in Glioblastoma cell lines based on database data. **(B)** The effect of BCL6 overexpression was detected by western blot in HS683 were transfected with BCL6^OE^ plasmid, and the overexpression effect of BCL6 was detected by western blot. **(C)** Cell viability readouts of NC and BCL6^OE^ cell lines derived from HS683 cells. **(D)** Clonogenic assay of NC and BCL6^OE^ cell lines derived from SHG44 cells. **(E)** The effect of BCL6 overexpression was detected by western blot in SHG44 were transfected with BCL6^OE^ plasmid, and the overexpression effect of BCL6 was detected by western blot. **(F)** Cell viability readouts of NC and BCL6^OE^ cell lines derived from SHG44 cells. **(G)** Clonogenic assay of NC and BCL6^OE^ cell lines derived from SHG44 cells.

**Figure S2**. **YK01 is a Novel and Potent of BCL6 Small Molecular Inhibitor.**

**(A)** Candidate compounds obtained through the preliminary screening of the HTRF drug screening system.**(B)** The synthesis scheme of YK01.

**Figure S3**.**YK01 suppresses transcriptional inhibitory activity of BCL6 *in vivo.***

**(A)** Flow cytometry detection of splenic GC B cells (B220+GL7+FAS+) percentage. **(B)** Body weights changes of each group on day14.

**Figure S4**.**FX1 and RI-BPI inhibits GBM cell proliferation.**

**(A)** MTS assays from 48 h FX1 treatment assessed the viability of multiple GBM cell lines. **(B)** MTS assays from 48 h RI-BPI treatment assessed the viability of multiple GBM cell lines. **(C)** IC_50_ values of FX1 and RI-BPI in GBM cells.

**Figure S5.YK01 can reactivate the BCL6-target genes.** **(A)** Changes in BCL6 target genes transcript abundance after different time with 2 μM YK01 treatment in U251 and U87-MG cell lines.

**Figure S6.The half-life time (t_1/2_) of BCL6 protein. (A)** U251 cell were treated with 20 µg/mL CHX to block translation. BCL6 levels were examined by immunoblot analysis every 2 hours for a total 16 hours. **(B)** U251 cell were treated with YK01 and 20 µg/mL CHX to block translation. BCL6 levels were examined by immunoblot analysis every 2 hours for a total 16 hours. **(C)** The t_1/2_ of BCL6 protein was calculated using the Graphpad Prism software.

**Figure S7. YK01 inhibits the activation of AKT and ERK in GBM cells.**The changes of p-AKT, t-AKT, p-ERK, t-ERK, p-PI3K, t-PI3K, p-p38 and t-p38with the concentration of YK01 were detected by western blot after 24 h treatment of U87-MG and U251 cells.

**Figure S8.** **Effect of TMZ in combination with YK01 on GBM cancer cell growth.** Combinational matrix shows proliferation after treatment with TMZ and YK01 in the U251, SF268 and U87-MG cell lines. Synergy scores were calculated using Synergyfinder software. HSA (highest single agent) algorithm was used to calculate synergy scores, and color keys indicate the scores in heatmaps.

**Figure S9. Drug administration induce apoptosis of GBM cells.** Apoptosis of U251 and U87-MG cells after treatment with TMZ and YK01 alone and in combination for 48 h at different concentrations. Statistical analysis of the proportion of apoptotic cells in U251 and U87-MG.
